# Genome-wide survey indicates diverse physiological roles of the barley (*Hordeum vulgare* L.) calcium-dependent protein kinase genes

**DOI:** 10.1038/s41598-017-05646-w

**Published:** 2017-07-13

**Authors:** Yunqiang Yang, Qiuli Wang, Qian Chen, Xin Yin, Min Qian, Xudong Sun, Yongping Yang

**Affiliations:** 1 Key Laboratory for Plant Diversity and Biogeography of East Asia, Kunming Institute of Botany, Chinese Academy of Science, Kunming, 650204 China; 2 Plant Germplasm and Genomics Center, Kunming Institute of Botany, Chinese Academy of Sciences, Kunming, 650201 China; 3 Institute of Tibetan Plateau Research at Kunming, Kunming Institute of Botany, Chinese Academy of Sciences, Kunming, 650201 China; 4 University of Chinese Academy of Sciences, Beijing, 100049 China

## Abstract

Calcium-dependent protein kinases (CDPKs) are crucial calcium sensors that play important roles in the regulation of plant growth and developmental processes, as well as protective responses to environmental stress. Here, we identified 28 *CDPK* genes from barley and cloned 5 new, full-length *CDPK* genes, *MLOC_58648a*, *MLOC_19618a*, *MLOC_71733a*, *AK249361a* and *MLOC_4965a*, using their expressed sequence tags. Phylogenetic and gene structural analyses revealed that the *CDPK* could be divided into four subgroups. Significant site-specific altered constraints and a high evolutionary rate may have contributed to the functional divergences among *CDPK* gene subfamilies. Expression profiles of different tissues and developmental stages suggested that several *CDPK* genes are involved in the functional development of plants. Different expression levels under a variety of abiotic stresses also indicated that the *CDPK* family underwent functional divergence during long-term evolution. Furthermore, several *CDPK* genes responded to single treatments and individual *CDPK* genes responded to multiple treatments, suggesting that barley *CDPK*s may be involved in mediating cross-talk among different signalling pathways. Our data provide an important foundation for the functional and evolutionary analyses of this important gene family in barley.

## Introduction

Plants have evolved a series of survival mechanisms to adapt to various environmental challenges, including high salinity, drought, low temperatures and pathogens stress. Calcium (Ca^2+^), as a ubiquitous secondary messenger, plays important roles in plants responses to these environmental stimuli^1, 2^. Under stress conditions, several Ca^2+^ sensors or Ca^2+^ binding proteins can sense changes in the cytoplasmic Ca^2+^ concentration and further regulate downstream genes to improve plant resistance^3^. Plant Ca^2+^ sensors or Ca^2+^ binding proteins are complex protein families that are divided into four major classes, calmodulin, calmodulin-like proteins, calcineurin B-like proteins and calcium-dependent protein kinases (CDPK)^3, 4^. Among the four protein families, CDPKs are unique because their protein kinase and calmodulin-like domains are present in a single polypeptide, resulting in Ca^2+^-binding and Ca^2+^-stimulated kinase activities within an independent protein product^3, 5^.

Typical *CDPK* family members have four distinct domains: a variable N-terminal domain, a protein kinase domain, an autoinhibitory domain and a calmodulin-like domain^3–5^. The N-terminal domains often contain palmitoylation or myristoylation sites, which are key to subcellular localization and function^6^, and show the highest sequence divergence among CDPK domains^5^. Moreover, the N-terminal domain is variable, with different lengths and amino acid compositions, and determines the specific function of the individual CDPKs^6, 7^. The protein kinase domain contains a catalytic domain for the binding of ATP and is adjacent to the autoinhibitory junction domain. The calmodulin-like domain contains one to four EF-hand structures for Ca^2+^ binding^4^. CDPK can be activated because the Ca^2+^ binding leads to a change in the protein’s conformation, altering the autoinhibitory domain^8^.

CDPKs play important roles in plant responses to various abiotic and biotic stresses, signalling the transduction of hormones^2, 4, 9, 10^. In *Arabidopsis*, *AtCPK4*/*11*/*3*/*6*/*21*, as positive regulators, were involved in tolerance to salt and drought stresses^11–13^. *CDPK* genes from other plants, such as *OsCDPK7*/*9*/*13* from rice^10, 14, 15^ and *ZmCDPK4*/*12* from maize^16, 17^, also have similar functions in the responses to salt and drought stresses. In addition, Ulloa *et al*.^18^ showed that jasmonic acid (JA) affects CDPK activity in plant responses to *Solanum tuberosum* infection^18^. The expression levels of some *CDPK* genes were increased after treatment with various plant hormones, including gibberellin, auxin and abscisic acid^16, 19^. However, other *CDPK* genes act as negative regulators. For example, *AtCDPK23* mutants show an increased tolerance to drought and salt stress, while overexpressing lines are more susceptible^20^. Thus, the functions of *CDPK* genes are complex in response to biotic or abiotic stress.

The genes encoding CDPKs form a multi-gene family and exist in different plant species. Genome-wide analyses have identified 34, 31, 26, 40, 30, 19, 27, 50, 41, 19, 31, 29 and 25 *CDPK* genes in *Arabidopsis* ^4^, rice^9^, wheat^21^, maize^22^, poplar^23^, grape^24^, cassava^25^, soybean^26^, cotton^27^, cucumber^28^, pepper^29^, tomato^30^ and canola^31^, respectively. Barley (*Hordeum vulgare* L.) was one of the first domesticated grains and is an important crop plants worldwide. It has a higher resistance to adverse environmental conditions than its close relative wheat^32^. Fedorowicz-Strońska *et al*.^33^ identified 27 *CDPK* genes from barley and analysed their expression levels under intensifying drought stress conditions^33^. However, 4 of the 27 barley *CDPK* genes, Hv*CDPK7*, *9*, *16* and *27*, were expressed sequence tags (ESTs), implying that the protein structures were not complete. Here, we identified 28 Hv*CDPK*s by a genome-wide analysis and cloned the full-length open reading frames (ORFs) of five novel Hv*CDPK* genes using their EST sequences from barley. Phylogenetic and gene structural analyses were performed to determine their evolutionary relationships. We further analysed the functional divergence of this gene family and the expression profiles of Hv*CDPK* genes in response to various abiotic stress conditions. Our results provide valuable information on the evolutionary history and the biological functions of the barley *CDPK* family.

## Results

### Identification of the Hv*CDPK* genes in barley

In this study, a genome-wide analysis of the *CDPK* gene family was performed using barley genome sequences found in the Ensembl Plants and PGSB-PlantsDB databases. A total of 25 proteins, including the alternatively spliced forms, had a conserved protein kinase domain and four EF-hand domains. Proteins that were similar to CDPK-related protein, calcium/calmodulin-dependent protein, and calcium and calcium/calmodulin-dependent protein kinases were removed (Table 1). Further, the 25 protein sequences were used as query to search the draft genome and predicted mRNAs resulted in no further hits. Among those with alternative splice variants (AK362157, AK358395 and AK366527, AK364859), we selected the longest variant for further analysis. Among the low-confidence genes of barley, 10 CDPK proteins were finally identified (Table 1). One of these proteins, MLOC_67965 from morex_contig_53987, was removed because the CDS was obviously terminated by a premature stop codon, resulting in a protein missing the EF-hand domain at its C-terminus, indicating that this gene may be a pseudogene.

**Table 1:** Summary information on *CDPK* genes from barley databases.

Gene Name^a^	Gene Name^b^	CDS length^a^	EST	PK	No. of EF hands	Contig name^a,b^	Chromosome^b^	Genomic location^b^
**Genes identified in HighNconfidence**								
MLOC_6391.1	MLOC_6391.1	1509	N	Y	4	morex_contig_137484	5	512,514,012N512,520,124(N)
AK373165	MLOC_77271.1	1692	N	Y	4	morex_contig_7499	2	481,951,258N481,956,106(+)
MLOC_59921.1	MLOC_59921.1	1563	N	Y	4	morex_contig_44036	5	120,076,425N120,080,953(+)
AK373462	MLOC_38029.1	1551	N	Y	4	morex_contig_2549571	4	265,459,716N265,461,542(+)
AK362157	NF	1674	N	Y	4	NF	N	NF
AK358395	MLOC_71042.1	1473	N	Y	4	morex_contig_5903	2	580,050,020N580,053,822(+)
AK366527	NF	1290	Y	Y	4	NF	N	NF
AK364859	MLOC_68114.1	1644	N	Y	4	morex_contig_54230	6	404,825,280N404,829,976(+)
MLOC_6934.1	MLOC_6934.1	1545	N	Y	4	morex_contig_138355	6HL	462,242N464,536(N)
MLOC_54510.1	MLOC_54510.1	1566	N	Y	4	morex_contig_39182	3	465,728,618N465,731,616(N)
MLOC_76003.1	MLOC_76003.1	1554	N	Y	4	morex_contig_69788	1	383,322,254N383,324,727(+)
MLOC_72770.1	MLOC_72770.1	1629	N	Y	4	morex_contig_62331	5	67,817,706N67,820,088(N)
AK363357	MLOC_32468.1	1584	N	Y	4	morex_contig_228852	1H	5,249,646N5,252,977(N)
MLOC_12765.1	MLOC_12765.1	1563	N	Y	4	morex_contig_1564051	3	409,133,979N409,137,786(+)
MLOC_79572.1	MLOC_79572.1	1599	N	Y	4	morex_contig_8570	2	495,289,533N495,294,149(+)
AK365481	MLOC_76047.1	1629	N	Y	4	morex_contig_6998	2	557,713,706N557,716,488(N)
AK371805	MLOC_43400.1	1653	N	Y	4	morex_contig_270067	3	499,954,458N499,961,096(+)
AK360154	MLOC_10811.1	1608	N	Y	4	morex_contig_1559810	5	528,572,674N528,577,167(N)
AK365058	MLOC_37356.2	1638	N	Y	4	morex_contig_2548222	2	246,285,070N246,288,483(+)
MLOC_21560.3	MLOC_21560.3	1680	N	Y	4	morex_contig_159616	5	263,661,148N263,667,423(+)
AK376018	MLOC_19040.1	1665	N	Y	4	morex_contig_1583223	5	457,260,839N457,262,164(N)
AK374710	MLOC_7568.2	1725	N	Y	4	morex_contig_139533	7	320,148,852N320,150,555(N)
MLOC_55774	MLOC_55774.3	1884	N	Y	4	morex_contig_40250	5	512,125,056N512,138,246(+)
AK372866	MLOC_72357.1	1524	N	Y	4	morex_contig_61497	6	30,208,994N30,211,144(N)
AK363630	NF	1521	N	Y	4	morex_contig_60290	NF	NF
**Genes identified in LowNconfidence**								
MLOC_58647.1	NF	717	Y	N	4	morex_contig_42856	5	66520687N66521405(N)
MLOC_58648.1	NF	858	Y	Y	0	morex_contig_42856	5	66514920N66515145(N)
AK249361.1	MLOC_39835.1	1065	Y	Y	4	morex_contig_2554997	4	521,672,389N521,676,123(N)
MLOC_67965.2	NF	750	Y	Y	0	morex_contig_53987	4	521,994,137N521,994,958(+)
MLOC_19618.2	MLOC_19618.2	714	Y	Y	0	morex_contig_1585699	1	405,806,477N405,806,934(+)
AK354090	NF	714	Y	N	4	morex_contig_53624	N	N
MLOC_4965.2	NF	513	Y	N	4	morex_contig_135729	4HS	614,167N615,057(N)
MLOC_71733.1	NF	921	Y	Y	4	morex_contig_60392	4	4:8966491N8967423(N)
MLOC_7896.1	NF	384	Y	Y	0	morex_contig_140474	4	4:8236725N8237105(N)
MLOC_71734.1	NF	351	Y	Y	0	morex_contig_60392	4	4:8968504N8968851(N)

NF, Not found; N, NO; Y, Yes.

^a^PGSB-PlantsDB database (ftp://ftpmips.helmholtzmuenchen.de/plants/barley/public_data/).

^b^Hordeum vulgare at Ensembl Plants (http://plants.ensembl.org/Hordeum_vulgare).

### Isolation of Hv*CDPK*s in the low-confidence genes of barley

To further determine full-length sequences of Hv*CDPK*s in low-confidence genes of barley, RACE tests were performed using primers designed from the EST sequences of Hv*CDPK*s, including *MLOC_58647*.*1*, *MLOC_58648*.*1*, *MLOC_19618*.*2*, *AK354090*, *AK249361*.*1*, *MLOC_4965*.*2*, *MLOC_71733*.*1*, *MLOC_71734*.*1* and *MLOC_7896*.*1* (Table 1). Two full-length CDSs of the *MLOC_58647* and *MLOC_58648* genes were cloned, and the gene sequences were aligned with the MAFFT 7.0 program. The two genes had the same nucleotide sequence, which was named *MLOC_58648a* (GenBank: KY008232; Figs S1a and S2). Further, chromosomal locational analyses showed that both *MLOC_58647*.*1* and *MLOC_58648*.*1* genes are located in the morex_contig_42856 on the chromosome 5 of barley (Table 1 and Fig. S3). Thus, the EST fragments of *MLOC_58647* and *MLOC_58648* may have been derived from the same gene. Meanwhile, similar results were found between *MLOC_19618*.*2* from morex_contig_1585699, which was located on the chromosome 1 of barley, and *AK354090* from morex_contig_53624, which has an unknown chromosomal location (Table 1). Thus, we predicted that *MLOC_19618*.*2* and *AK354090* represented the same *CDPK* gene, designated *MLOC_19618a* (GenBank: KY008233; Figs S1b and S2 and Tables 1 and 2), and previous studies may have contained an assembly error. The full-length cDNAs of *MLOC_71733*.*1*, *MLOC_71734*.*1* and *MLOC_7896*.*1* were cloned, and similar results were also found for these three EST sequences. Here, the novel *CDPK* gene was named *MLOC_71733a* (GenBank: KY008234; Fig. S1c and Table 2). In addition, the full-length cDNAs of *AK249361* and *MLOC_4965*.*2* were amplified from the EST sequence, and named *AK249361a* (GenBank: KY008235) and *MLOC_4965a* (GenBank: KY008236), respectively (Figs S1d,e and S2 and Table 2). The primer sequences are shown in Table S1. Finally, we identified 28 barley *CDPK*s, including 23 *CDPK*s from the Ensembl Plants and PGSB-PlantsDB databases (high-confidence genes) and 5 novel full-length *CDPK* genes (Fig. S1 and Table 2).

**Table 2:** CDPK genes identified and cloned from barley.

Name	Locus Id^a^	Protein ID^**b**^	GenBank	Number of amino acids:	MW (Da)	Pl	GRAVY	No. of EF hands	N-Myr	N-Pal	N-Term
HvCPK1	MLOC_12765.1	MLOC_12765.1	ACA63885	520	58634.6	5.85	−0.486	4	Y	Y	MGNRT
HvCPK2	MLOC_54510.1	MLOC_54510.1	BAK06618	521	57503.4	5.59	−0.402	4	Y	Y	MGNCC
HvCPK3	AK371805	MLOC_43400.1	BAK03003	550	61449.1	6.63	−0.429	4	Y	Y	MGNCC
HvCPK4	AK372866	MLOC_72357.1	BAJ86092	507	57038.2	8.68	−0.419	4	Y	Y	MGACL
HvCPK5	AK364859	MLOC_68114.1	BAJ96062	547	60401.6	5.39	−0.274	4	Y	Y	MGNTC
HvCPK6	MLOC_6934.1	MLOC_6934.1	NF	514	56507.9	5.2	−0.272	4	NF	NF	MGRGA
HvCPK7	AK249361a*	AK249361.1	KY008235	548	61004.3	5.15	−0.278	4	NF	NF	MGNQN
HvCPK8	AK360154	MLOC_10811.1	BAJ91363	535	60056.3	6.31	−0.475	4	Y	Y	MGNCC
HvCPK9	MLOC_71733a*	NF	KY008234	565	62908.7	9.11	−0.424	4	Y	Y	MGNAC
HvCPK10	NF	MLOC_55774.3	BAJ88027	627	68244.9	5.92	−0.275	4	NF	NF	MGNTS
HvCPK11	MLOC_6391.1	MLOC_6391.1	NF	502	55753.9	5.19	−0.22	4	NF	Y	MAPVA
HvCPK12	AK365481	MLOC_76047.2	BAJ96684	542	60360.1	6.1	−0.402	4	Y	Y	MGNCF
HvCPK13	AK362157	MLOC_71042.2	BAJ93361	557	61417.8	5.55	−0.315	4	Y	Y	MGNAC
HvCPK14	MLOC_76003.1	MLOC_76003.1	BAK07881	517	57397.6	6.31	−0.365	4	Y	NF	MGMCC
HvCPK15	AK363357	MLOC_32468.	BAJ94561	527	59523.7	6.1	−0.554	4	Y	Y	MGGRA
HvCPK16	MLOC_19618a*	BAJ85309	KY008233	457	51802.3	5.94	−0.345	4	NF	Y	MGRGA
HvCPK17	AK373165	MLOC_77271.1	NF	563	61866.2	5.21	−0.294	4	Y	Y	MGNTC
HvCPK18	AK363630	BAJ86849	BAJ86849	506	56714.7	6.75	−0.393	4	Y	Y	MGLCT
HvCPK19	MLOC_79572.1	MLOC_79572.1	BAJ99143	532	59376.6	5.63	−0.41	4	Y	Y	MGQCC
HvCPK20	AK365058	MLOC_37356.1	BAJ96261	545	61434	6.49	−0.488	4	Y	Y	MGNCC
HvCPK21	AK374710	MLOC_7568.1	BAK05906	574	63555.9	8.44	−0.471	4	NF	Y	MGGCY
HvCPK22	AK376018	BAK08155	BAK07213	554	61555.7	5.41	−0.486	4	Y	Y	MGGCY
HvCPK24	AK373462	NF	BAK04659	516	56912.6	5.3	−0.278	4	NF	Y	MQPDA
HvCPK25	MLOC_72770.1	MLOC_72770.1	BAK06838	542	59709.4	4.99	−0.448	4	Y	Y	MGQCC
HvCPK26	MLOC_4965a*	NF	KY008236	532	58730.8	5.79	−0.419	4	Y	Y	MGQRC
HvCPK27	MLOC_58648a*	NF	KY008232	623	68786	5.68	−0.41	4	Y	Y	MGNVC
HvCPK28	MLOC_59921.1	MLOC_59921.1	NF	520	57377.4	5.53	−0.276	4	NF	Y	MQPDP
HvCPK29	MLOC_21560.3	MLOC_21560.1	BAK05737	559	62755.8	5.87	−0.307	4	Y	Y	MGNCC

*Newly cloned CDPK gene; ^a^PGSB-PlantsDB database; ^b^Hordeum vulgare at Ensembl Plants; NF, Not found.

### Characterization and chromosomal location analysis of Hv*CDPK*s

The barley genome has 28 CDPK genes, all containing four EF-hand domains and coding sequences of 457–627 amino acids (Table 2). The 28 CDPK proteins, designated as HvCDPK1 to HvCDPK28, according to Fedorowicz-Strońska^33^ and their homology to *CDPK* genes in rice. Eight, HvCDPK6, 7, 10, 11, 16, 21, 25 and 28, of the 28 HvCDPK proteins do not contain a myristoylation motif CDPK; however, 24 of the 28 HvCDPK proteins (excluding HvCDPK6, 7, 10 and 14) have palmitoylation sites (Table 2). The chromosomal localizations of the *CDPK* genes were analysed based on the Ensembl Plants database. Chromosome 5 contained the maximum number of *CDPK* genes, which was eight (Fig. S3), followed by chromosome 2, which had 6 *CDPK* genes. Chromosomes 3 and 4 each contained 3 *CDPK* genes, while chromosomes 1 and 6 each had 2 genes, respectively. Chromosome 7 contained 1 *CDPK* gene (Fig. S3). Hv*CDPK6*, *15*, *18*, *23* and *26* may be located in gap regions because their chromosomal locations could not be identified.

### Phylogenetic relationships and gene structural analyses of the Hv*CDPK*s

**Figure 1:**
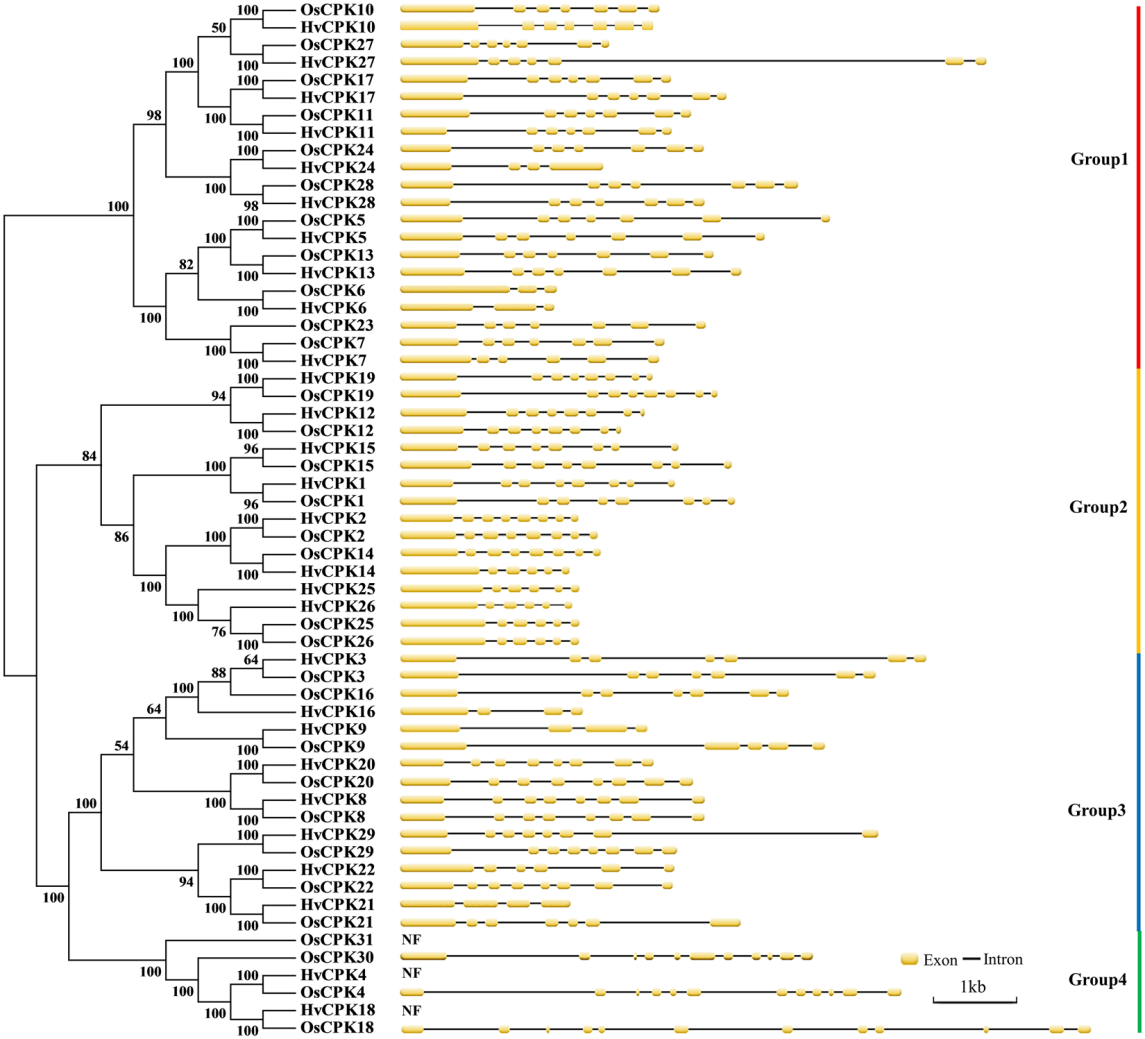
Phylogenetic relationships among rice and barley CDPK proteins. The molecular phylogeny was constructed from a complete protein sequence alignment of CDPKs from rice and barley using the neighbour-joining method with a bootstrapping analysis (1,000 replicates). The numbers beside the branches indicate bootstrap values. The four subgroups designated from 1 to 4 are displayed in different colours.

**Figure 2:**
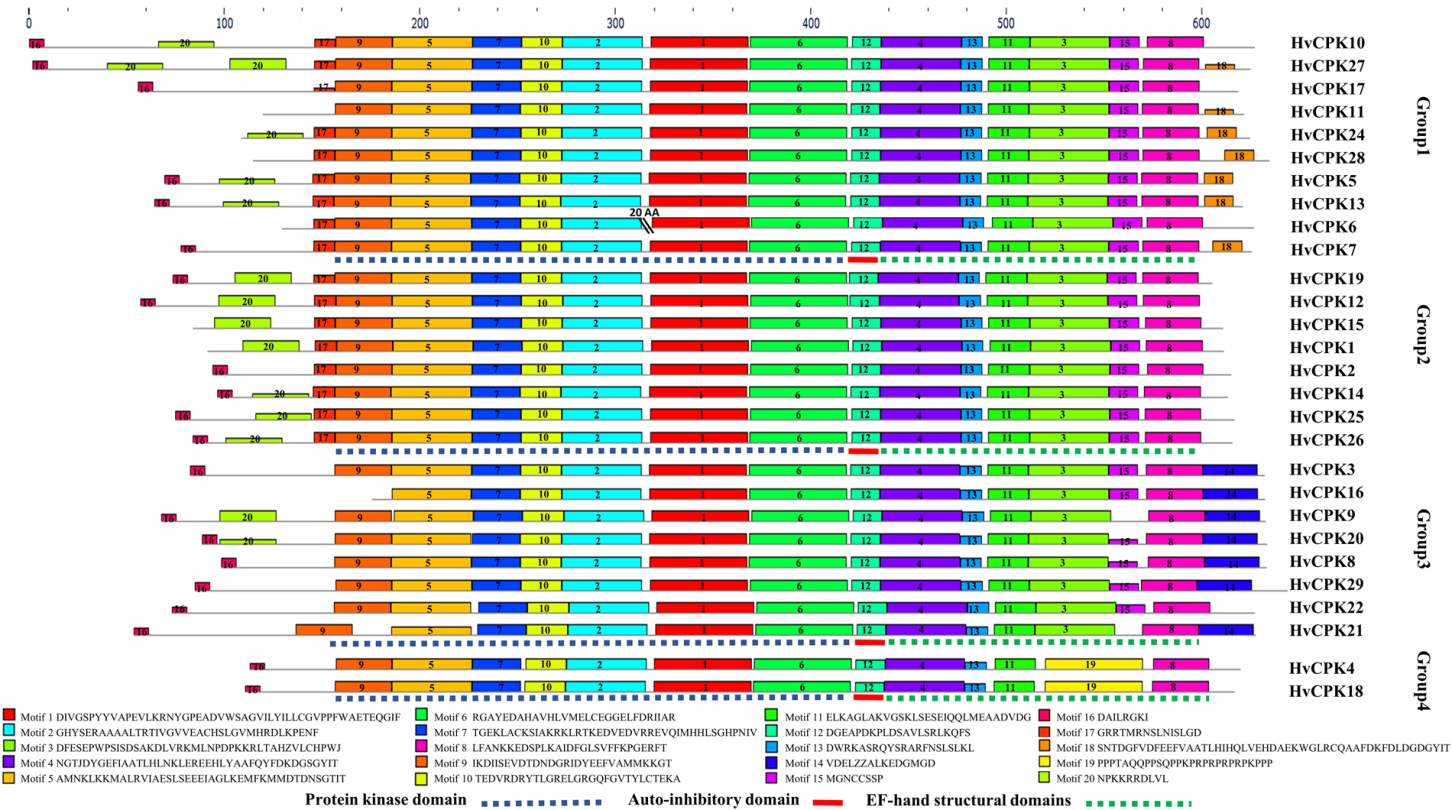
Motif organizations in 28 Hv*CDPK*s. The conserved motifs were detected using the MEME online tool (http://meme.sdsc.edu/meme/intro.html) and SMART (http://smart.embl-heidelberg.de/). The conserved protein kinase domain, auto-inhibitory domain and EF-hand structural domain are denoted by blue, red and green underlined, respectively.

To examine the phylogenetic relationships among the Hv*CDPK* genes and other *CDPK*s in plants, a neighbour-joining tree was constructed using CDPK protein sequences from barley and rice. Based on our phylogenetic results, the CDPKs were divided into four major groups (Fig. 1), consistent with previous reports^23, 26, 29^. The exon/intron structure of the CDPK family was analysed by comparing full-length cDNAs with their corresponding genomic sequences. The number of exons determined for members of the *CDPK* gene family ranged from 3 in HvCDPK6 and Os*CPK*6 to 12 in Os*CPK*4 and 18. The *CDPK* genes of Groups 1, 2 and 3 showed similarities in CDSs and splicing patterns, having six to seven exons, except HvCDPK6 and Os*CPK*6, which had three exons. HvCDPK9, 16, 21 and 24 contained four exons, as did Os*CPK*9. In addition, a similar exon/intron structure was found between homologous *CDPK* genes in barley and rice (Fig. 1). Thus, most CDPKs in the same cluster appear to have very similar exon-intron structures, strongly supporting their close evolutionary relationships and representing gene family expansion from ancient paralogs or multiple origins of gene ancestry. The *CDPK* genes of Group 4 had 11 to 12 exons, but we could not determine the number of exons of OsCPK31 or HvCDPK4 and 18 owing to a lack of genomic information. In addition, 20 conserved motifs within the barley *CDPK* genes were identified using online MEME tools (Figs 2 and S4). As mentioned above, phylogenetic analyses broadly divided the *CDPK* genes into four major groups. Eleven of the motifs (motif 5, 7, 10, 2, 1, 6, 12, 4, 13, 11 and 8) were shared by all of the CDPK proteins. Meanwhile, the conserved gene structures revealed unique motifs among groups (Fig. 2). For instance, motif 18 can be found in Group 1, motif 14 can be found in Group 3, and motif 19 can be found in Group 4. These results further illustrate that the function of the CDPK proteins within the same groups are similar, but there may be functional divergences between different groups.

### Evolutionary history of the barley *CDPK* gene family

Phylogenetic analyses also showed that barley CDPKs were highly similar to their best rice matches, and 26 pairs of barley-rice CDPK proteins were putative paralogs with percentage identities ranging from 72.70% to 93.62% (Fig. 1 and Table 3). Thus, we also estimated T of 26 pairs of barley-rice putative CDPK paralogous proteins by measuring the Ks and Ka mutation rates using an r of 6.5 × 10^−9^ mutations per Ks site per year. The estimated Ts for barley-rice CDPK orthologs were between 30.20 to 63.20 million years ago (MYA) following the divergence of barley and rice (50–70 MYA)^34^. The average T of pairs of barley-rice CDPK was calculated at ~40 MYA with a standard deviation of 8 MYA. This rough dating provides an approximate time of the divergence of barley and rice *CDPK* genes. In addition, the Ka/Ks (ω) value was calculated for each pair of *CDPK* orthologous genes. The ω values for all of the putative CDPK paralogs having mean values of 0.076 were less than 1, suggesting that the 26 pairs of barley-rice CDPK proteins are under a strong purifying selection pressure (Table 3). However, three pairs of barley-rice CDPK proteins, HvCDPK9/OsCPK9 (ω = 0.1986), HvCDPK21/OsCPK21 (ω = 0.1927) and HvCDPK22/OsCPK22 (ω = 0.1813), had relatively large ω values, indicating that they may have evolved rapidly from the last common ancestor.

**Table 3:** Estimated divergence time between bayley–rice CPK orthologs.

Seq1	Seq2	Identity (%)	Ks	Ka	ω	T(MYA)
HvCPK1	OsCPK1	89.27	0.3928	0.0327	0.0832	30.2
HvCPK2	OsCPK2	91.36	0.4322	0.0241	0.0558	33.2
HvCPK3	OsCPK3	92.38	0.5176	0.0193	0.0373	39.8
HvCPK4	OsCPK4	86.59	0.5382	0.0221	0.0411	41.4
HvCPK5	OsCPK5	89.09	0.5212	0.0264	0.0507	40.1
HvCPK6	OsCPK6	72.70	0.7647	0.104	0.136	58.8
HvCPK7	OsCPK7	76.92	0.5636	0.0662	0.1175	43.4
HvCPK8	OsCPK8	84.60	0.5373	0.0607	0.113	41.3
HvCPK9	OsCPK9	81.14	0.4078	0.081	0.1986	31.4
HvCPK10	OsCPK10	84.40	0.652	0.0276	0.0423	50.2
HvCPK11	OsCPK11	79.51	0.4912	0.0376	0.0765	37.8
HvCPK12	OsCPK12	80.15	0.5575	0.0504	0.0904	42.9
HvCPK13	OsCPK13	90.16	0.4355	0.0188	0.0432	33.5
HvCPK14	OsCPK14	86.81	0.4271	0.035	0.0819	32.9
HvCPK15	OsCPK15	82.48	0.5196	0.0367	0.0706	40
HvCPK16	OsCPK16	80.80	0.4225	0.0155	0.0367	32.5
HvCPK17	OsCPK17	76.22	0.6148	0.0323	0.0525	47.3
HvCPK18	OsCPK18	89.06	0.4468	0.0285	0.0638	34.4
HvCPK19	OsCPK19	93.62	0.5689	0.02	0.0352	43.8
HvCPK20	OsCPK20	87.61	0.4278	0.0273	0.0638	32.9
HvCPK21	OsCPK21	74.83	0.4038	0.0732	0.1813	31.1
HvCPK22	OsCPK22	73.18	0.5318	0.1025	0.1927	40.9
HvCPK24	OsCPK24	91.86	0.5011	0.0192	0.0383	38.5
HvCPK27	OsCPK27	80.22	0.4907	0.0275	0.056	37.7
HvCPK28	OsCPK28	88.61	0.5773	0.028	0.0485	44.4
HvCPK29	OsCPK29	78.38	0.8221	0.0619	0.0753	63.2

### Analysis of functional divergence

To determine the adaptive functional diversification of the CDPK family, an analysis of type-I functional divergence between CDPK subgroups was executed using the DIVERGE 2.0 program, which evaluates the shifted evolutionary rate and altered amino acid properties. The type-I functional divergence of amino acid sites was compared between the conservative and non-conservative subgroups. As shown in Table 4, the coefficient of type-I functional divergence values varied from 0.052 to 0.352 in CDPK subgroups 1/2, 1/3, 1/4, 2/3, 2/4 and 3/4. These observations indicate that there were significant site-specific altered selective constraints on most members of the CDPK family, leading to group-specific functional evolution after diversification.

**Table 4:** Functional divergence estimated between CDPK subfamilies.

Comparison	θ^a^	SE^b^	LRT^c^	P^d^	Qk > 0.70^e^
Group 1/Group 2	0.052	0.0339	2.3528	<0.05	1
Group 1/Group 3	0.2424	0.0373	42.2984	<0.05	15
Group 1/Group 4	0.352	0.0522	45.5503	<0.05	34
Group 2/Group 3	0.1856	0.0404	21.0668	<0.05	6
Group 2/Group 4	0.2656	0.0662	16.1063	<0.05	7
Group 3/Group 4	0.3462	0.0559	38.396	<0.05	12

^a^θ is the coefficient of type I functional divergence between two groups.

^b^SE: standard error.

^c^LRT is a likelihood ratio test.

^d^The significance level (P value) is computed using Fisher’s transformation.

^e^N (0.7) indicate the number of divergent residues when the cut-off value was 0.7.

Based on the posterior probability of each comparison, the evolutionary rates at specific amino acid sites were predicted to identify sites of functional divergence among the CDPK subfamilies. To reduce false positives, a cut-off value of 0.7 was applied to identify type-I functional divergence-related residues in the CDPK subfamilies. When the CDPK sequences in the four classes were compared, 34 critical amino acid sites were predicted for Group 1/4 pairs. Thus, the functional divergence analysis suggested that, because of the differences in the numbers and distributions of predicted sites of functional divergence within each pair, the *CDPK* genes may be divergent from each other in their functions. In addition, we also found higher coefficient of type-I functional divergence values in CDPK subgroups 1/4 (0.352). Thus, a higher evolutionary rate may have prompted the functional divergence of *CDPK* genes and the evolution of new functions after divergence.

### Expression profiles of barley *CDPK* genes at different developmental stages

**Figure 3:**
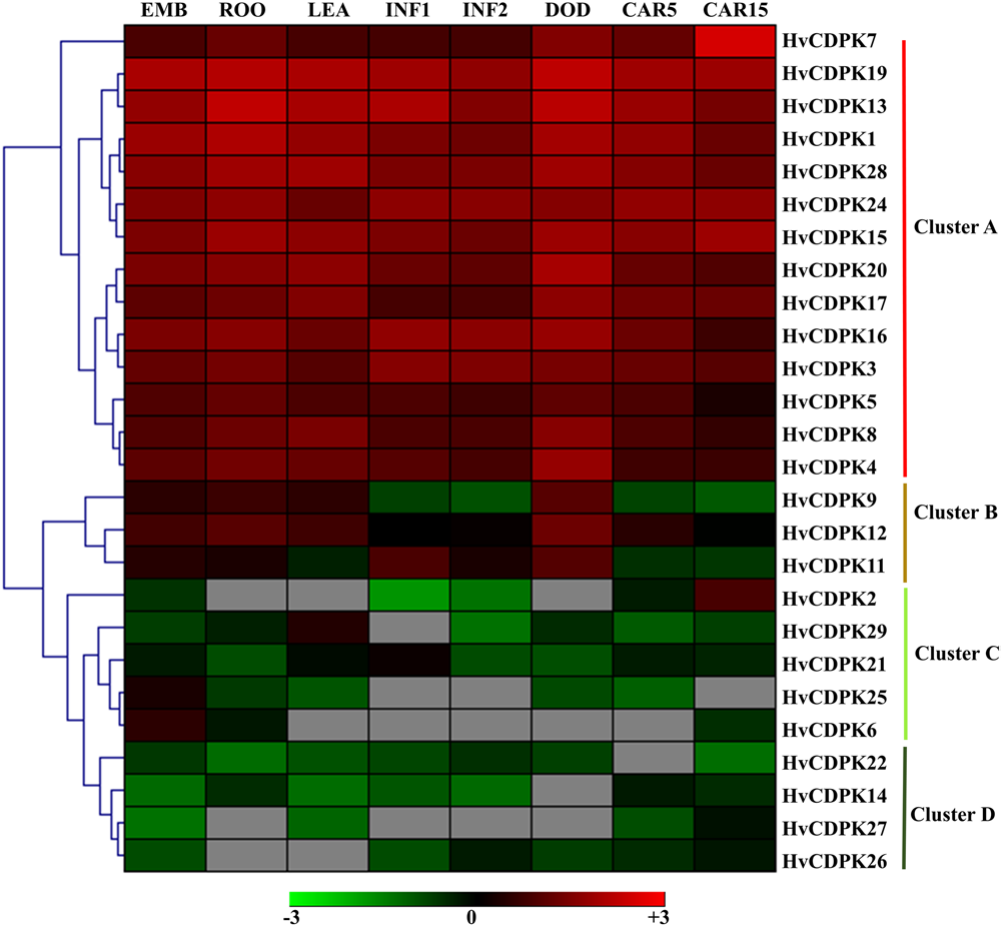
Expression profiles of barley *CDPK* genes in different tissues and developmental stages. Dynamic expression profiles using the FPKMs of the Hv*CDPK* genes in different tissues and development periods. FPKM values (log2 ratio) were gene-wise normalized and hierarchically clustered using Genesis software. Genes highly or weakly expressed are colored red and green, respectively, and gray represents the FPKM value of 0. EMB; four-day-old embryos dissected from germinating grains, ROO; roots from the seedlings (10-cm shoot stage), LEA; shoots from the seedlings (10-cm shoot stage), INF1; young developing inflorescences (5 mm), INF2; developing inflorescences (1–1.5 cm), NOD; developing tillers at the six-leaf stage (3rd internode), CAR5; developing grains, with bracts removed (5 days post-anthesis), CAR15; developing grains, with bracts removed (15 days post-anthesis).

Gene functions can usually be predicted by their expression profile information. The expression levels of *CDPK* genes were analysed using publicly available RNA-sequence data from eight different barley tissues and developmental stages^32^. Twenty-six of the 28 Hv*CDPK* genes’ expression levels were obtained based on FPKM values, but FPKM data for Hv*CDPK10* and *18* were not found. We selected the FPKM data of the longest EST fragments of the newly acquired low-confidence Hv*CDPK* genes, including Hv*CDPK16*, *9*, *7*, *27* and *26*, to analyse their expression levels (Fig. S5). A heat map was created through the hierarchical clustering of the gene expression profiles of 26 Hv*CDPK* genes, and these could be divided into four clusters: Cluster A, B, C and D (Fig. 3). The 14 *CDPK* genes in Cluster A were highly expressed in eight different tissues and developmental stages, implying that Cluster A’s *CDPK* genes may play important roles in barley development. Cluster B’s *CDPK* genes were expressed most highly in the 3rd internodes of the six-leaf stage seedlings. Among Cluster C’s *CDPK* genes, Hv*CDPK25* and Hv*CDPK6* were almost exclusively expressed in embryos, Hv*CDPK21* was highly expressed in shoots and inflorescences, Hv*CDPK29* was highly expressed in shoots, and Hv*CDPK2* was highly expressed in grain, suggesting that they may be involved in the growth and development of these organs. However, four *CDPK* genes from Cluster D had very low expression levels throughout the sample set.

### Expression profiles of Hv*CDPK* genes during MeJA treatment

**Figure 4:**
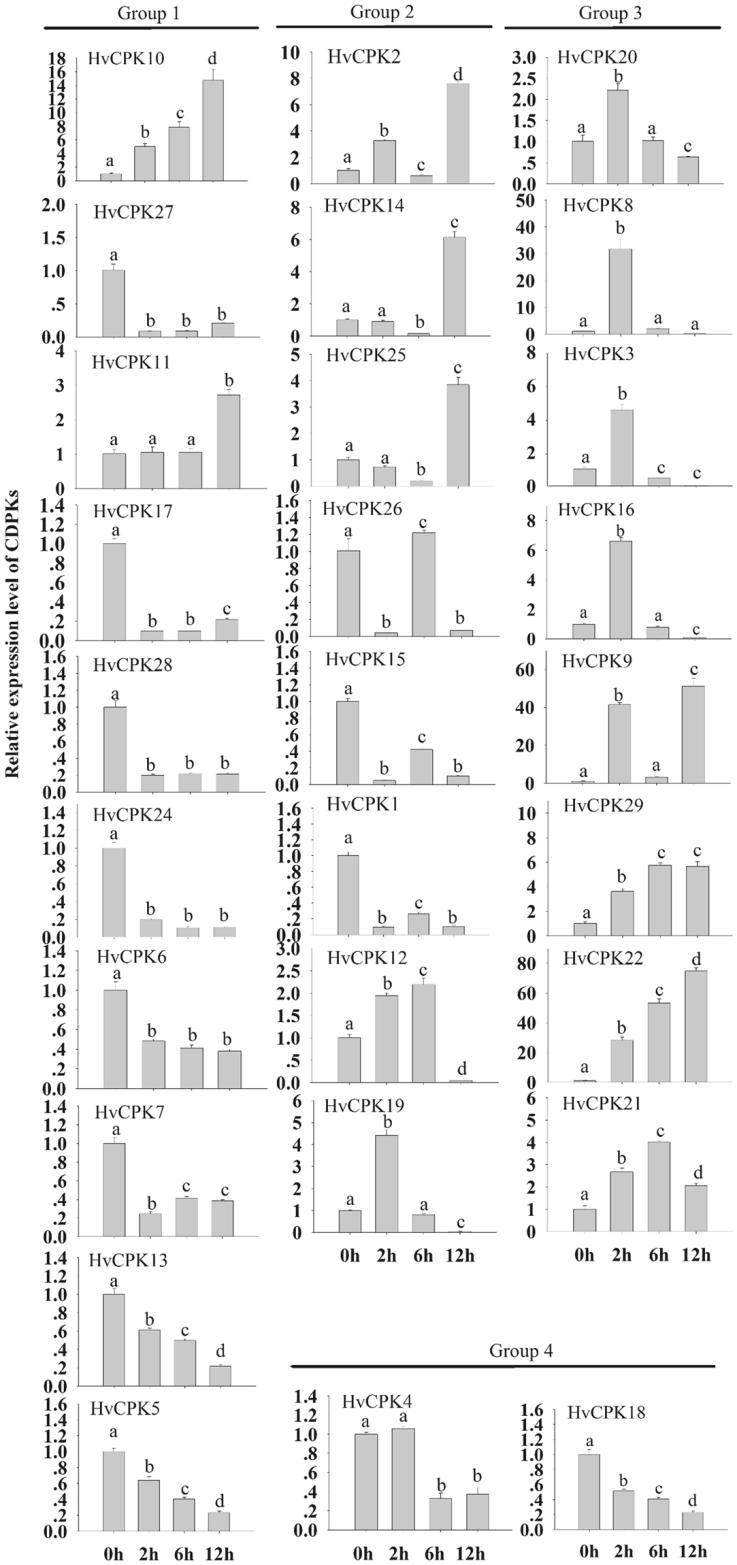
Expression profiles of the *HvCDPK* genes during MeJA treatment. qPCR analyses were performed, and expression values were calculated using the 2^−ΔΔCT^ method. Data are mean values ± SE obtained from three replicates. Different letters within a column indicate a significant difference (P < 0.05; Tukey’s test).

Biotic and abiotic stresses are important restrictive factors affecting plant growth and development. The identification and study of plant resistance genes can be addressed at the molecular level using gene expression profiles to reveal the mechanisms of plant resistance. To analyse the responses of Hv*CDPK*s to MeJA treatment, the expression levels of 28 Hv*CDPK*s were examined by qRT-PCR. As shown in Fig. 4, the expression levels of 28 Hv*CDPK*s showed different tendencies. Further, we divided the Hv*CDPK*s genes into four subfamilies according to how they clustered in the phylogenetic tree to test for potential functional divergences among the four groups (Fig. 4). In Group 1, 8 of the 10 Hv*CDPK* genes, with the exceptions of Hv*CDPK10* and Hv*CDPK11*, showed lower expression levels in MeJA-treated plants than in controls, and a similar trend was found in Group 4. However, transcripts of Hv*CDPK*s in Group 2, except Hv*CDPK25* and Hv*CDPK1*, increased when plants were treated with MeJA. Meanwhile, the expression levels of all the Hv*CDPK* genes in Group 3 were greater than those of the untreated plants after 2 h of MeJA treatment. In particular, expression levels of Hv*CDPK22* were upregulated by more than 70-fold (12 h) compared with their respective levels at 0 h. These results indicated that the 28 Hv*CDPK*s had different response patterns to the same stimulus (Fig. 4). Additionally, combined with analyses of functional divergence (Table 4), this suggested that the barley Hv*CDPK* genes in the four subgroups may have different evolutionary histories, and probably novel functional divergence and adaptation.

### Expression profiles of the Hv*CDPK* genes under abiotic stress

**Figure 5:**
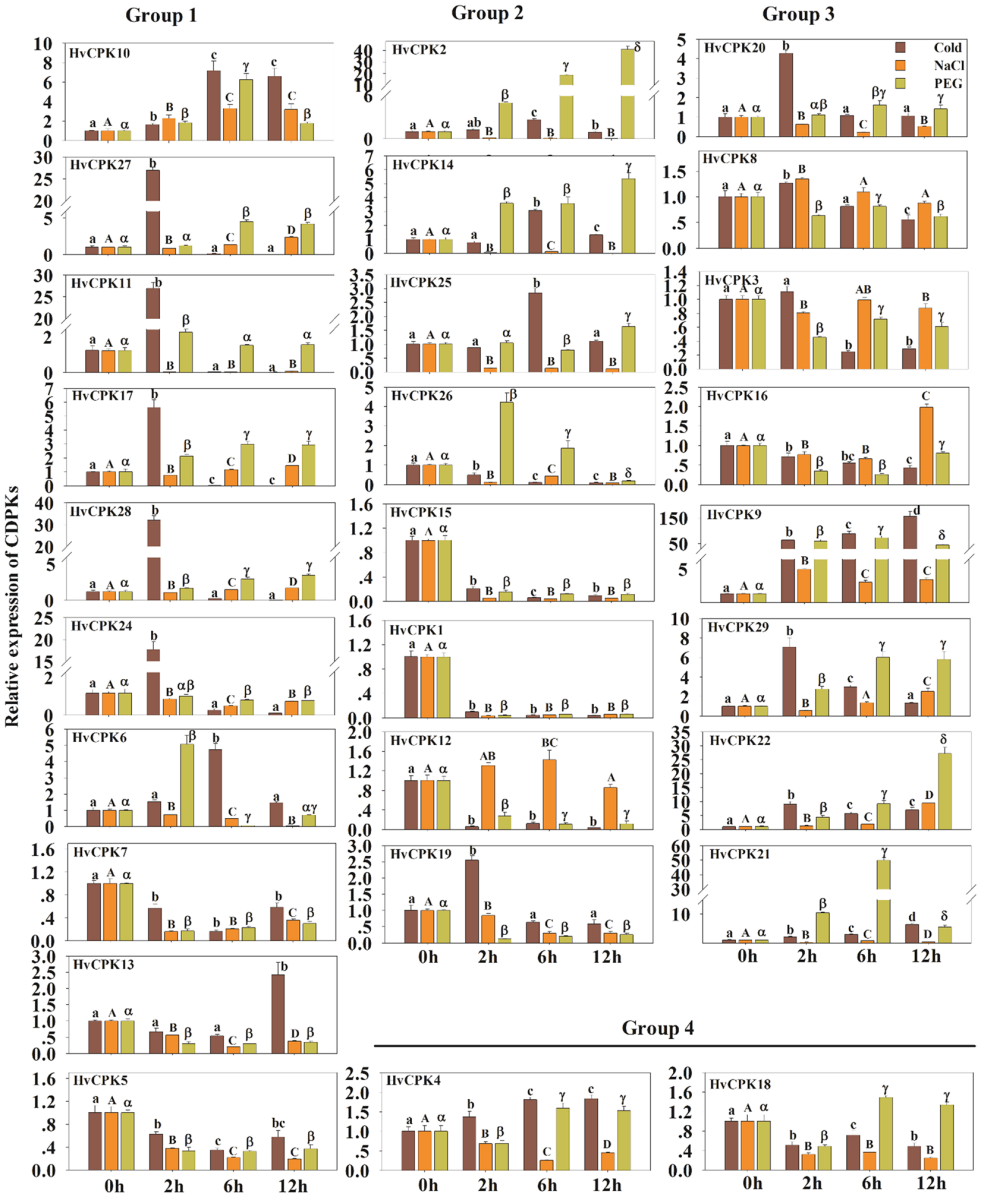
Expression profiles of the *HvCDPK* genes under cold, salt and PEG stress conditions. Data are mean values ± SE obtained from three replicates. Different letters within a column indicate a significant difference (P < 0.05; Tukey’s test).

**Figure 6:**
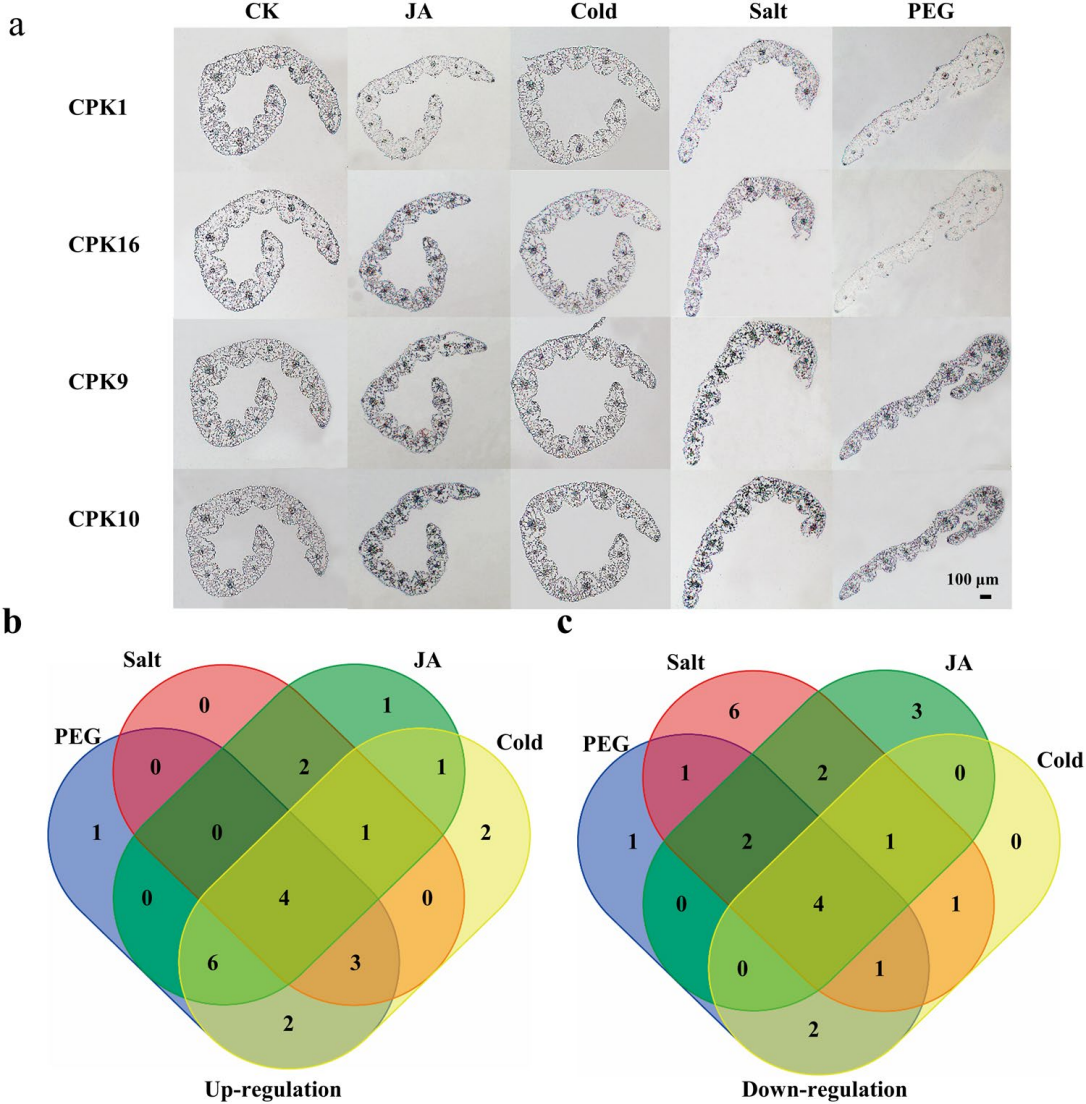
Localization of CDPK transcripts and cross-talk among different signalling pathways. (**a**) Longitudinal section of leaves probed with DIG-labeled antisense Hv*CDPK1*, *16*, *9* and *10* transcripts. Bar = 100 μm. Venn diagram showing the overlap of HvCDPK gene up-regulation (**b**) and down regulation (**c**) expression in response to MeJA, cold, salt and PEG treatments.

In plants, many *CDPK*s play important roles in responses to cold, salt and drought^1, 35^. To further evaluate the possible functional divergence of Hv*CDPK* genes during abiotic stress, we determined the expression pattern of Hv*CDPK* genes in response to cold (4 °C), NaCl (250 mM) and PEG (15%) treatments. Under cold stress, 5 of 10 Hv*CDPK* genes in Group 1 (Hv*CDPK27*, *11*, *17*, *28* and *24*) were upregulated at 2 h after treatment. Hv*CDPK 6*, *10* and *13* expression levels peaked at 6 h, 6 h and 12 h after treatment, respectively. Two down-regulated genes (Hv*CDPK7* and *25*) were also identified after treatment (Fig. 5). In Group 2, four Hv*CDPK* genes, Hv*CDPK2*, *14*, *25* and *19*, were up-regulated, whereas Hv*CDPK26*, *15*, *1* and *12* were significantly down-regulated. All of the Hv*CDPK* genes in Group 3, except for Hv*CDPK3* and *16*, were up-regulated by cold stress. In addition, the transcript abundance of Hv*CDPK4* in Group 4 was up-regulated, while Hv*CDPK18* was down-regulated. Under salt stress, four Hv*CDPK* genes (Hv*CDPK10*, *27*, *17* and *28*) in Group 1, one Hv*CDPK* gene (Hv*CDPK12*) in Group 2 and five Hv*CDPK* genes (Hv*CDPK8*, *16*, *9*, *29* and *22*) in Group 3 were up-regulated, whereas the rest of the Hv*CDPK* genes were down-regulated, including Hv*CDPK4* and *18* in Group 4 (Fig. 5). In response to the PEG treatment, six Hv*CDPK* genes in Group1, four Hv*CDPK* genes in Group 2, five Hv*CDPK* genes in Group 3 and all of the Hv*CDPK* genes in Group 4 were up-regulated (Fig. 5). In addition, by *in situ* hybridization, Hv*CDPK1*, *16*, *9* and *10* transcripts were detected in the leaves of barley under different treatments. As shown in Fig. 6a, strong signal of Hv*CDPK1*, *16*, *9* and *10* genes were found in the control samples, respectively. Compared with the control, Hv*CDPK9* and *10* showed stronger signal, but Hv*CDPK1* showed weaker signal after JA, cold, salt and drought treatments. Moreover, Hv*CDPK16* were up-regulated following JA treatment. These changes in signal of genes expressing tissueregions were consistent with the changes in the overall expression level demonstrated by qRT-PCR. Thus, several Hv*CDPK* genes responded to a treatment, and individual Hv*CDPK*s responded to multiple treatments (Fig. 6b and c), suggesting that barley *CDPK*s may be involved in mediating cross-talk among different signalling pathways.

## Discussion

*CDPK*s have been found only in plants and some protozoans, and play vital roles in plant growth and development, as well as abiotic and biotic stress responses. *CDPK*s have been identified in the genome information of several plants, including *Arabidopsis* ^4^, rice^36^, wheat^21^, cassava^25^, grape^24^, poplar^23^, maize^22^, canola^31^, cucumber^28^, pepper^29^ and soybean^26^. The draft barley genome provided valuable new information for the identification and research on *CDPK* genes or/and the gene family in barley. However, determining the number of *CDPK* gene family members also posed a challenge because of low-confidence barley genes, which were identified as potential gene fragments. Until recently, 27 members of the *CDPK* gene family had been identified in barley. However, 4 of the 27 CDPK protein structures were incomplete^33^. In this study, we investigated members of the CDPK family based on barley whole-genome data, including Ensembl Plants and PGSB-PlantsDB databases. In total, 25 full-length *CDPK* genes were obtained from the barley genome, including two alternatively spliced transcripts (Tables 1 and 2). In addition, five full-length cDNA were cloned using *CDPK* gene fragments from the low-confidence genes of the PGSB-PlantsDB barley database (Figs S1 and S2). Finally, a total of 28 CDPK sequences from barley were predicted and/or cloned in this study (Fig. 1). All 28 CDPK proteins contain protein kinase domains and 4 EF-hand calcium-binding domains, which is less than the 31 members reported in rice^36^ and more than the 26 members in wheat^21^. Research has shown that in total, 84% of the barley genome is mobile elements or other repeat structures^32^. In our research, tandem duplications of *CDPK* genes were observed in Chromosome 5, including Hv*CDPK27*/Hv*CDPK25* and Hv*CDPK10*/Hv*CDPK11* (Fig. S3 and Tables 1 and 2), which may be involved in the expansion of the *CDPK* gene family in barley. However, whether the *CDPK* gene family has undergone expansion by segmental genome duplication events in barley still needs to be confirmed.

In terms of biochemical properties, most of the CDPK proteins were slightly acidic, with isoelectric points (pI) in the 5–7 range^28^. Meanwhile, a few CDPK proteins from angiosperms, which were mainly found within Group 4, had basic pIs of 8 or more^3^. Barley CDPK proteins in Groups 1 and 2 have similar pIs, ranging from 4.99 to 6.31. However, pIs of 9 and 8 were predicted in Group 3 (Hv*CDPK9* and *21*, Table 2) and Group 4 (Hv*CDPK4*, Table 2), respectively. The most divergent homologs within the *CDPK* gene family indicate that a basic pI may correlate with a specific subcellular localization or function of Group 4 *CDPK*s^3, 28^. Here, Hv*CDPK9* and *21* in Group 3, the pIs of 9.11 and 8.44, respectively, may contribute to their functional specificity because different residues have different pI values. However, this still needs to be better understood.

CDPK proteins with less than four EF-hands have been reported in both monocotyledon and dicotyledoneae, such as nine of 34 in *Arabidopsis* ^4^, four of 25 in canola^31^, one of 19 in cucumber^28^, four of 29 in tomato^30^, four of 50 in soybean^26^ and one of 31 in rice^9^, two of 19 in grape^24^, three of 30 in poplar^23^, four of 40 in maize^22^, respectively. Our investigation of gene structure showed that all 28 barley *CDPK* genes contain four EF-hands (Table 2). This result is similar to that of pepper^26^, as a dicotyledonous plant, all 31 CDPKs contain four EF-hands. Here, we did not get regular patterns about CDPK proteins with less than four EF-hands between monocotyledon and dicotyledoneae. In addition, 20 conserved motifs were identified within the barley CDPK proteins by analysing their structural diversification (Fig. 2). The structural divergence of the core proteins correlates with sequence divergence^37^. Our analyses revealed that most of the CDPK proteins include the conserved protein kinase domain, which is mainly composed of motifs 9, 5, 7, 10, 2, 1 and 6 (Figs 2 and S4), and a conserved auto-inhibitory domain (motif 12). However, the loss of motifs 3 and 15 were observed in the Group 4 (HvCDPK4 and 18; Fig. 2). Furthermore, motifs 4, 13, 11, 3, 15 and 8 form the four EF-hand structural domains in Groups 1–3, and motifs 4, 13, 11, 19 and 8 form the four EF-hand structural domains in Group 4. Meanwhile, motifs 18 and 14 appeared to be specific to Group 1 (except HvCDPK10, 17 and 16) and Group 3 (except HvCDPK21), respectively. Within each group, sequence divergences were found mainly in the N-terminal domains of CDPK proteins contained myristoylation and palmitoylation sites, which may bind CDPK proteins to membranes and/or promote protein-protein interactions^6, 7, 38, 39^. Research has shown that CDPKs N-terminal protein undergo first modifications by acylations corresponding to N-a-acetylation and N-myristoylation, and follow by further reversible modifications such as phosphorylation or further acylations such as palmitoylation for membrane anchoring^6, 7^. In addition, because N-terminus lacks a cysteine residues, AtCPK3 can only be N-myristoylated, but not palmitoylated, and this effect correlated with nonspecific membrane localization and gene function of AtCPK3^13^. In the present study, eight of CDPKs lacked myristoylation sites and four of CDPKs lacked palmitoylation sites (Table 2). However, future biochemical studies are needed to unravel the lack of N-myristoylation and/or palmitoylation site may cause subcellular localization difference and potential functional divergence of HvCDPK protein family.

Gene divergence plays an important role in the evolution of novel functions^28, 40^. Changes in the amino acid sites of the conserved region may result in the functional divergence of a protein family member^41^. In our study, the divergences in protein sequences among different subgroups indicated that the CDPK paralogues may have a variety of physiological functions. Therefore, we investigated the Type-I functional divergence between the gene groups of the *CDPK* family using DIVERGE software, evaluating changes in the evolutionary rate and amino acid properties^41^. The functional divergence analysis showed that the coefficients of the functional divergence values were more than 0 (Table 4), indicating that site-specific altered selective constraints on most members of the *CDPK* family led to group-specific functional evolution after diversification. The functional divergences of *CDPK* genes based on gene expression information have been reported in other plants, such as grapevine^42^ and cucumber^28^. To further determine the functional divergence of the barley *CDPK* family, we analysed the expression levels of *CDPK* genes in different tissues and developmental stages under different stress treatments. In non-stressed barley plants, most of the *CDPK*s in clusters A and B were expressed in different tissues and developmental stages (Fig. 3). Five *CDPK* genes in Cluster C showed tissue-specific expression. Similarly *Capsicum annuum CDPK22* was expressed constitutively in roots, and Ca*CDPK2*, *3*, *4* and *31* were expressed specifically in flowers^29^. Moreover, some *CDPK* genes, such as *Glycine max CDPK28* and *50*, showed very low expression levels in different tissues and developmental stages, include young leaves, roots, flowers, pod, seeds and nodules^26^. In the present study, Cluster D showed similar expression patterns, with low expression levels across all eight samplings (Fig. 3). The duplicated genes may have different evolutionary fates, and one of the duplicates has a divergent expression pattern^43^. In the gene pair Hv*CDPK27* and Hv*CDPK25*, tandem duplicates on Chromosome 5 (Fig. S3), had different transcriptional levels in different tissues and developmental stages (Fig. 3). Interestingly, Hv*CDPK25* was down-regulated, whereas Hv*CDPK27* was up-regulated under cold stress (Fig. 5), and Hv*CDPK25* was up-regulated, whereas Hv*CDPK27* was down-regulated under salt stress (Fig. 5). These results indicated that the homologs of *CDPK*s have evolved more specified functions through gene divergence to help the plant meet a broader array of lineage-specific requirements^26, 28^.

*CDPK* genes are generally induced by different hormones and various types of stress^22, 23^. In the present study, barley *CDPK* genes from the same subgroup exhibited similar expression patterns during a MeJA treatment (Fig. 4). In addition, *CDPK* genes were differentially expressed after cold, salt and drought stresses (Fig. 5). Thus, cross-talk may have helped regulate the signalling network of Hv*CDPK* genes being expressed under various types of stress. Four genes, Hv*CDPK10*, *9*, *29* and *22*, which were up-regulated in response to MeJA, cold, salt and drought stresses (Fig. 6a). In addition, the results of *in situ* hybridization analysis also confirmed that the expression level of Hv*CDPK9* and *10* were higher in MeJA, cold, salt and drought-treated plants than in controls (Fig. 6a). Until now, there have been no reports on the Hv*CDPK9* (MLOC_71733.1) and Hv*CDPK10* (MLOC_55774.3) gene’s resistance to environmental stress in barley. Based on the phylogenetic tree, Hv*CDPK9* was clustered with rice *OsCPK9*, which was induced by abscisic acid, PEG, NaCl and rice *blast* tolerance^10, 44^, and Hv*CDPK10* was clustered with rice *OsCPK10*, which could enhance rice resistance against *Magnaporthe grisea* when overexpressed^45^. Genes with the same functions are often closely related^46, 47^, indicating that Hv*CDPK9* and *10* play important roles in different signal transduction pathways and in the adaptation of barley to changeable environments and stresses. In addition, the expression levels of the Hv*CDPK29* and *22* genes were also significantly increased after cold, salt, drought and MeJA treatments. The specific roles of Hv*CDPK29* and *22* in barley resistance to environmental stress need further study.

In summary, we identified 28 CDPK genes in the barley genome. Significant site-specific altered constraints and a higher evolutionary rate may have contributed to the functional divergence of HvCDPKs genes. Different expression levels of HvCDPKs genes under a variety of abiotic stresses also indicated that the barley CDPK gene family has functionally diverged during long-term evolution. Further, Hv*CDPK9* and *10* were up-regulated by *in situ* hybridization analysis in barely response to MeJA, cold, salt and drought treatment, indicating that Hv*CDPK9* and *10* play vital role in enhancing barley tolerance to changeable environments and stresses. Our analyses provide an important foundation for understanding the potential roles of HvCDPKs in regulating barley responses to biotic and abiotic stresses. Further research on barley CDPKs’ biological functions are needed to determine their effects on plant adaptations to adverse conditions and the underlying mechanisms.

## Methods

### Identification of the *CDPK* gene family in barley

The coding (CDS) and predicted protein sequences of barley were obtained from the Ensembl Plants (http://plants.ensembl.org/Hordeum_vulgare/Info/Index) and Plant Genome and Systems Biology (PGSB)-PlantsDB (ftp://ftpmips.helmholtzmuenchen.de/plants/barley/public_data/) databases, and included 26,159 high-confidence genes and 53,220 low-confidence genes^32^. The domains and functional sites of all of the protein were examined using the domain analysis programs ps_scan.pl. All of the protein sequences containing protein kinase domains (PS50011) and EF-hand calcium-binding domains (PS50222) were extracted and used to search against the GenBank non-redundant (Nr) protein database. Among the low-confidence genes of barley from the PGSB-PlantsDB database, all of the protein sequences with protein kinase domains (PS50011) or EF-hand calcium-binding domains (PS50222) were also extracted and used to search against the GenBank Nr protein database. Finally, based on the domain analysis, we removed the sequences of CDPK-related protein, calcium/calmodulin-dependent protein, and calcium and calcium/calmodulin-dependent protein kinases, and the remaining proteins were considered as barley CDPKs.

### RNA isolation and full-length Hv*CDPK* cloning

Total RNA was isolated using TRIzol reagent (Invitrogen, Carlsbad, CA, USA) from barley samples. RNA quality was characterized initially on an agarose gel and a NanoDrop ND1000 spectrophotometer (NanoDrop Technologies, Wilmington, DE, USA), following the manufacturer’s instructions. After the total RNA was isolated, DNA-free total RNA (5 μg) was used for first-strand cDNA synthesis using Superscript III reverse transcriptase (Invitrogen) according to the manufacturer’s instructions. Then, rapid amplification of cDNA ends (RACE) was conducted using a SMART RACE cDNA Amplification Kit (BD Biosciences Clontech, USA), following the manufacturer’s instructions. To obtain the full-length Hv*CDPK* CDSs from a barley sample, primers were designed based on the sequencing results (3′ and 5′ RACE). The full-length Hv*CDPK* CDSs were amplified in a total volume of 50 μL containing 3.0 U Taq DNA polymerase (Takara), 2 mM MgCl_2_, 1 × PCR buffer (Takara), 0.2 mM of dNTP (Takara), 0.8 µM of each primer and 1 µL of cDNA. The PCR program was as follows: 4 min at 94 °C, 33 cycles of denaturation for 30 s at 94 °C, annealing for 30 s at 52 °C and elongation for 120 s at 72 °C, followed by a final extension of 10 min at 72 °C. The PCR products were transformed and amplified in *Escherichia coli* DH5α cells. The positive transformants were selected through blue/white screening and then sequenced.

### Sequence and phylogenetic analyses and divergence time estimation

The full-length barley *CDPK* cDNA sequences from low-confidence genes were translated using EidtSeq program of DNASTAR software. The EF-hand and protein kinase motifs were predicted by SMART (http://smart.embl-heidelberg.de/)^48^. The molecular weight (MW), theoretical pI and grand average of hydropathicity (GRAVY) were calculated using the ProtParam tool of ExPaSy (http://web.expasy.org/protparam/)^49^. Myristoylaton motifs were predicted using PlantsP (http://plantsp.genomics.purdue.edu/myrist.html). Palmitoylation and N-terminal acylation predictions were performed using CSS-Palm 3.0^50^ and NetAcet 1.0^51^ software, respectively. The MEME program was used to search for conserved motifs in the barley candidate CDPK protein sequences^52^. A total of 31 full-length rice^9^ and barley CDPK protein sequences were aligned using the MAFFT 7.0 program, and a phylogenetic reconstruction was performed by MEGA7 software using the neighbour-joining method^53^. Bootstrap values were estimated (with 1,000 replicates) to assess the relative support for each branch. Gene intron/exon structures were analysed using GSDS (http://gsds.cbi.pku.edu.cn/)^54^. The global alignment of CDPK gene pairs were performed by the MAFFT 7.0 program. The aligned sequences were subsequently transferred into original cDNA sequences using the PAL2NAL web server (http://www.bork.embl.de/pal2nal/). Synonymous (Ks) and nonsynonymous (Ka) substitution rates were estimated by the codeml program of PAML4^55^. The divergence time (T) of barley and rice *CDPK* gene pairs were calculated using the formula T = Ks/2r, where r represents the divergence rate of 6.5 × 10^−9^ mutations per Ks site per year^56^.

### Functional divergence analysis

Among the CDPK subgroups, the coefficients of type-I functional divergence were calculated to estimate the level of functional divergence and predict amino acid residues responsible for functional differences between any two clusters following the methods of Gu^41^ using the DINERGE 2.0 software according to the instruction manual^41^.

### The differential expression profile of *CDPK* genes

Expression levels of CDPK genes were estimated using fragments per kilobase of exon model per million mapped reads (FPKM) values of eight different tissues and developmental stages. FPKM values were obtained from the barley genome explorer (http://apex.ipk-gatersleben.de/apex/f?p=284:10), and the normalization and hierarchical clustering analysis of gene expression patterns were performed based on Pearson coefficients with average linkage using the Genesis software (version 1.7.1)^57^.

### Plant material and abiotic stress

Seeds of barley were cleaned and surface-sterilized in a solution of 2% sodium hypochlorite for 15 min, rinsed five times in sterilized water and germinated in plastic trays lined with wet paper towels for 36 h in the dark at 23 °C. The seedlings were grown in soil pots and 1/4 Hoagland’s nutrient solution under controlled conditions (28 °C day/25 °C night cycle, 200 mmol photons m^−2^ s^−1^ light intensity and 75–80% relative humidity). After two weeks of germination, seedlings were exposed to cold (4 °C), methyl jasmonate (MeJA) (100 μm) or high-salinity stress (250 mM NaCl), or treated with 15% (w/v) polyethylene glycol (PEG). Plants were harvested during the treatments at 2, 6 and 12 h, with 0 h as the control. All of the samples were kept at −80 °C until used for RNA isolation.

### Analysis of quantitative real-time PCR (qRT-PCR)

qRT-PCRs of Hv*CDPK*s were performed as previously described^58^. All of the primers were designed by Primer-BLAST (http://www.ncbi.nlm.nih.gov/tools/primer-blast/index.cgi?LINK_LOC=BlastHome), applying the following parameters: 150–200 bp of PCR product size, Nr database, 57–63 °C primer melting temperatures (Tm), and *H*. *vulgare* subsp. vulgare (taxid:112509) for ‘Organism’. All of the PCR reactions were performed under the following conditions: 40 cycles of 5 s at 95 °C, 30 s at 60 °C, and 15 s at 72 °C. The barley *ubiquitin* gene was used as the control (GenBank: AAA62699). The primers are shown in Table S2.

### *In situ* hybridization

Plants were grown and treated as described above. Leaves were harvested at 0 d (as control) and 2 d of the treatments and fixed in 50 mM sodium phosphate buffer (pH 7.4) containing 4% (w/v) paraformaldehyde, respectively. Histological examinations and RNA (CDPKs) *in situ* hybridization analyses were performed as described by Mira *et al*.^59^. 500–600 bp fragment from CDPKs coding region were amplified by PCR using specific primers, respectively (Table S3). Then, the PCR fragments were cloned into pGEM-T-easy vector (Promega) and linearized using Nco1 or Sal1 before being used for synthesis of DIG-UTP-labeled sense or antisense probes, respectively. The probes were synthesized following the procedure described in the DIG Application Manual and were detected with single nitroblue tetrazolium/5-bromo-4-chloro-3-indolyl phosphate (NBT/BCIP) staining.

## Electronic supplementary material

Supplementary Information
