# Supplementary material for: Genome-wide survey indicates diverse physiological roles of the barley (Hordeum vulgare L.) calcium-dependent protein kinase genes
Source: Sci Rep. 2017 Jul 13;7:5306. doi: 10.1038/s41598-017-05646-w (PMC5509701; doi:10.1038/s41598-017-05646-w)
Supplement: Supplementary file 1 — Supplementary Information [file 41598_2017_5646_MOESM1_ESM.pdf]

Supplementary Figures and Tables

Genome-wide survey indicates diverse physiological roles of the barley (*Hordeum vulgare* L.) calcium-dependent protein kinase genes

Yunqiang Yang[1,2,3], Qiuli Wang[1,2,3], Qian Chen[1,2,3], Xin Yin[1,2,3,4], Min Qian[1,2,3], Xudong Sun[1,2,3*], Yongping Yang[1,2,3*]

[1]Key Laboratory for Plant Diversity and Biogeography of East Asia, Kunming Institute of Botany, Chinese Academy of Science, Kunming, 650204, China

[2]Plant Germplasm and Genomics Center, Kunming Institute of Botany, Chinese Academy of Sciences, Kunming 650201, China

[3]Institute of Tibetan Plateau Research at Kunming, Kunming Institute of Botany, Chinese Academy of Sciences, Kunming 650201, China

[4]University of Chinese Academy of Sciences, Beijing 100049, China

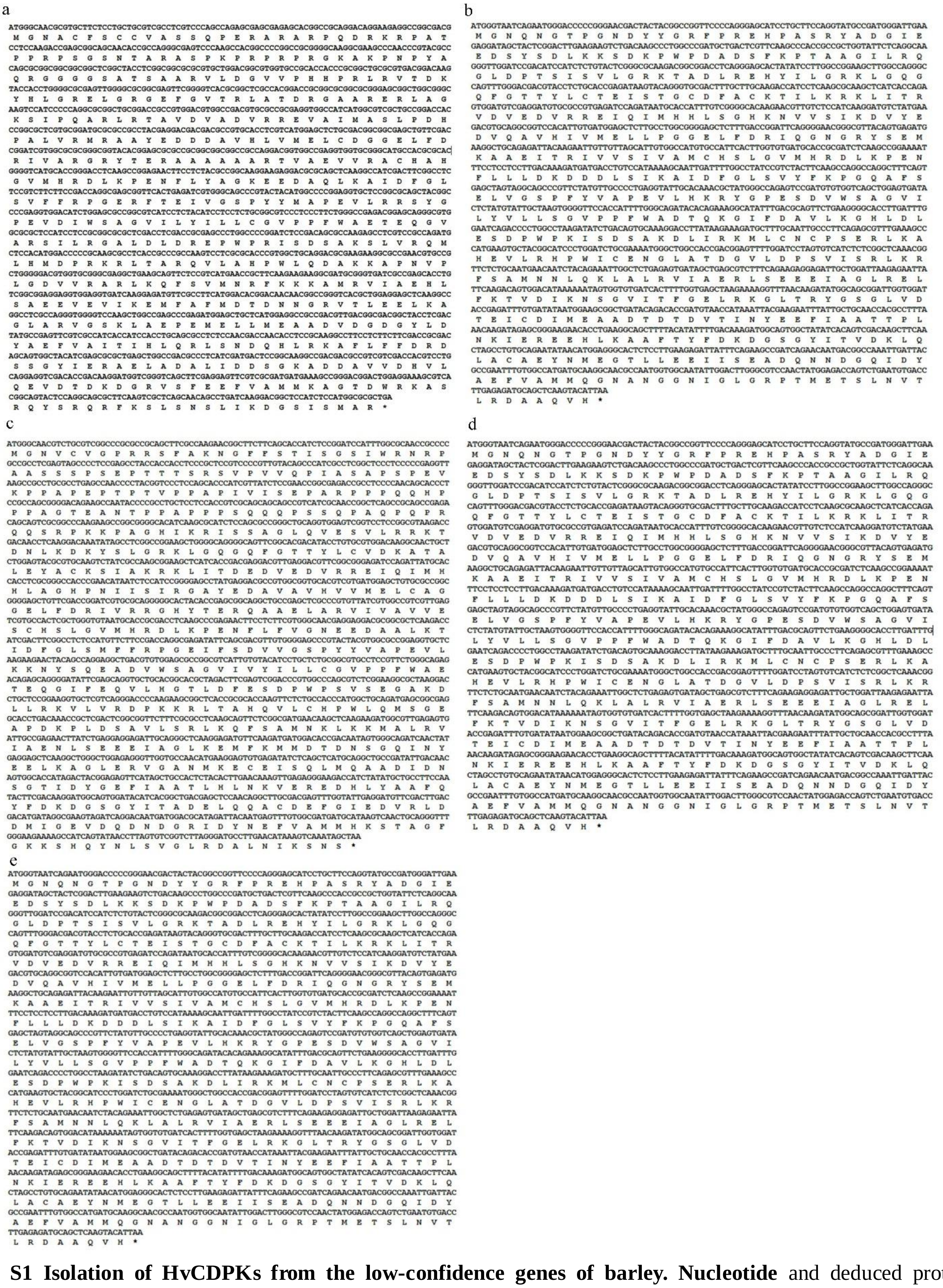

Fig. S1 Isolation of HvCDPKs from the low-confidence genes of barley. Nucleotide and deduced protein sequences of *MLOC_58648a* (GenBank accession: KY008232) (**a**), (**b**) *MLOC_19618a* (GenBank accession: KY008233), (**c**) *MLOC_71733a* (GenBank accession: KY008234), (**d**) *AK249361a* (GenBank accession: KY008235) and (**e**) *MLOC_4965a* (GenBank accession: KY008236).

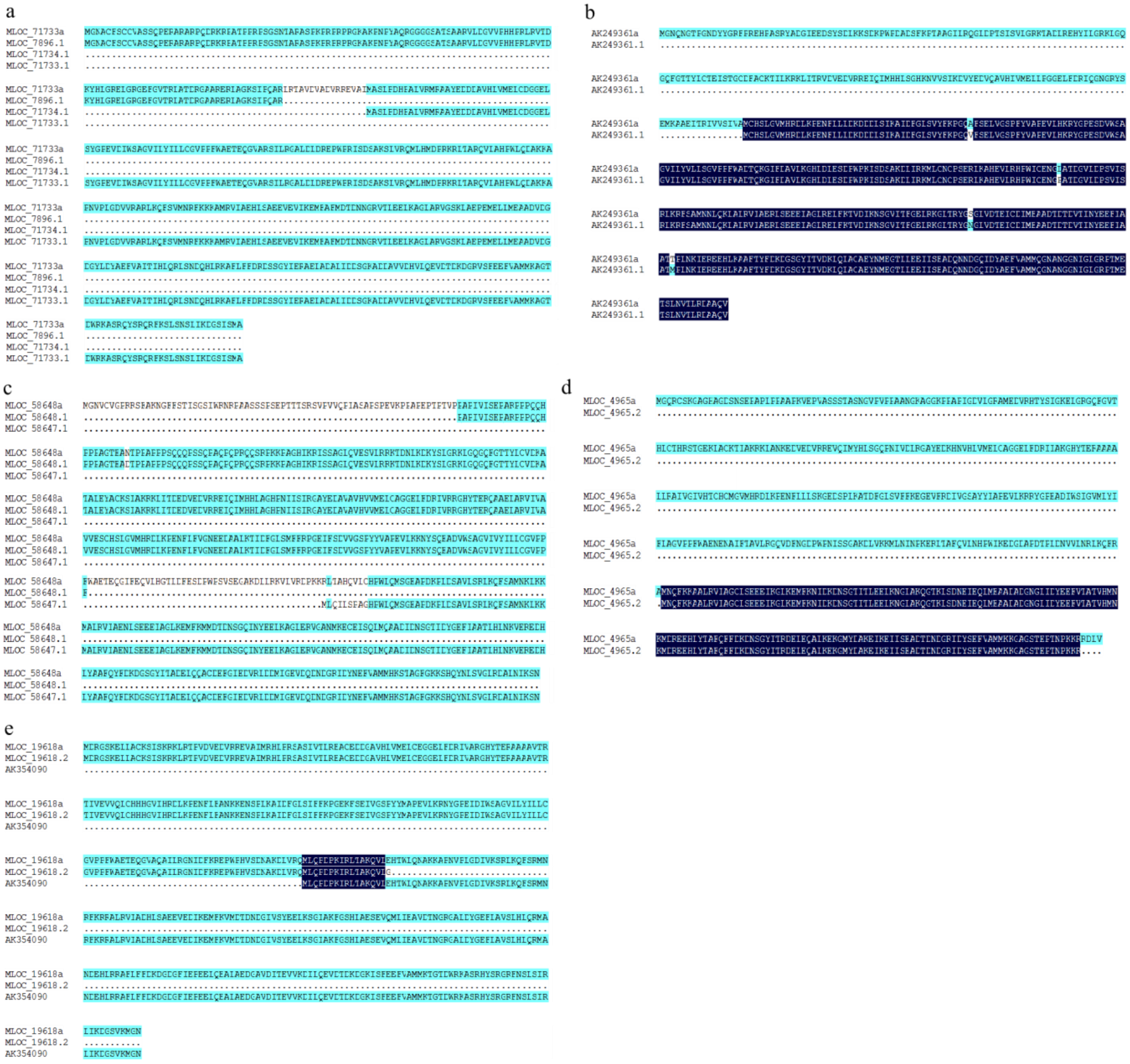

Fig. S2 Multiple sequence alignment of *MLOC_58648a*(a), *MLOC_19618a*(b), *MLOC_71733a*(c), *AK249361a*(d) and *MLOC_4965a*(e) with their EST sequences.

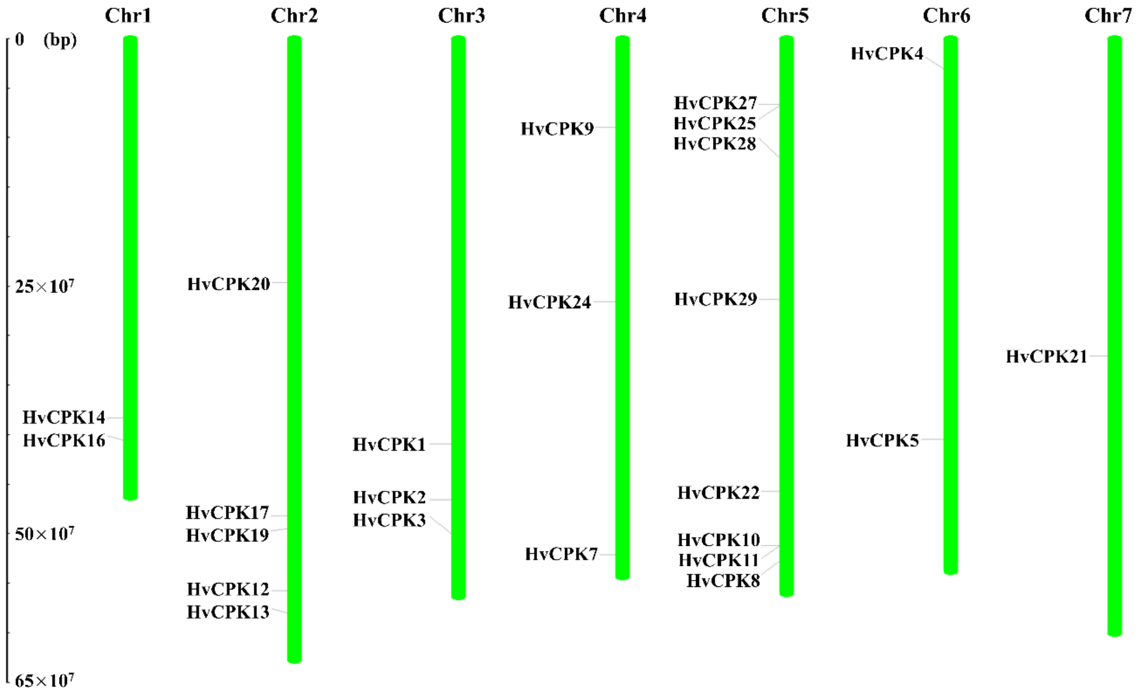

Fig. S3 Chromosomal locations of Hv*CDPK*genes in barley.

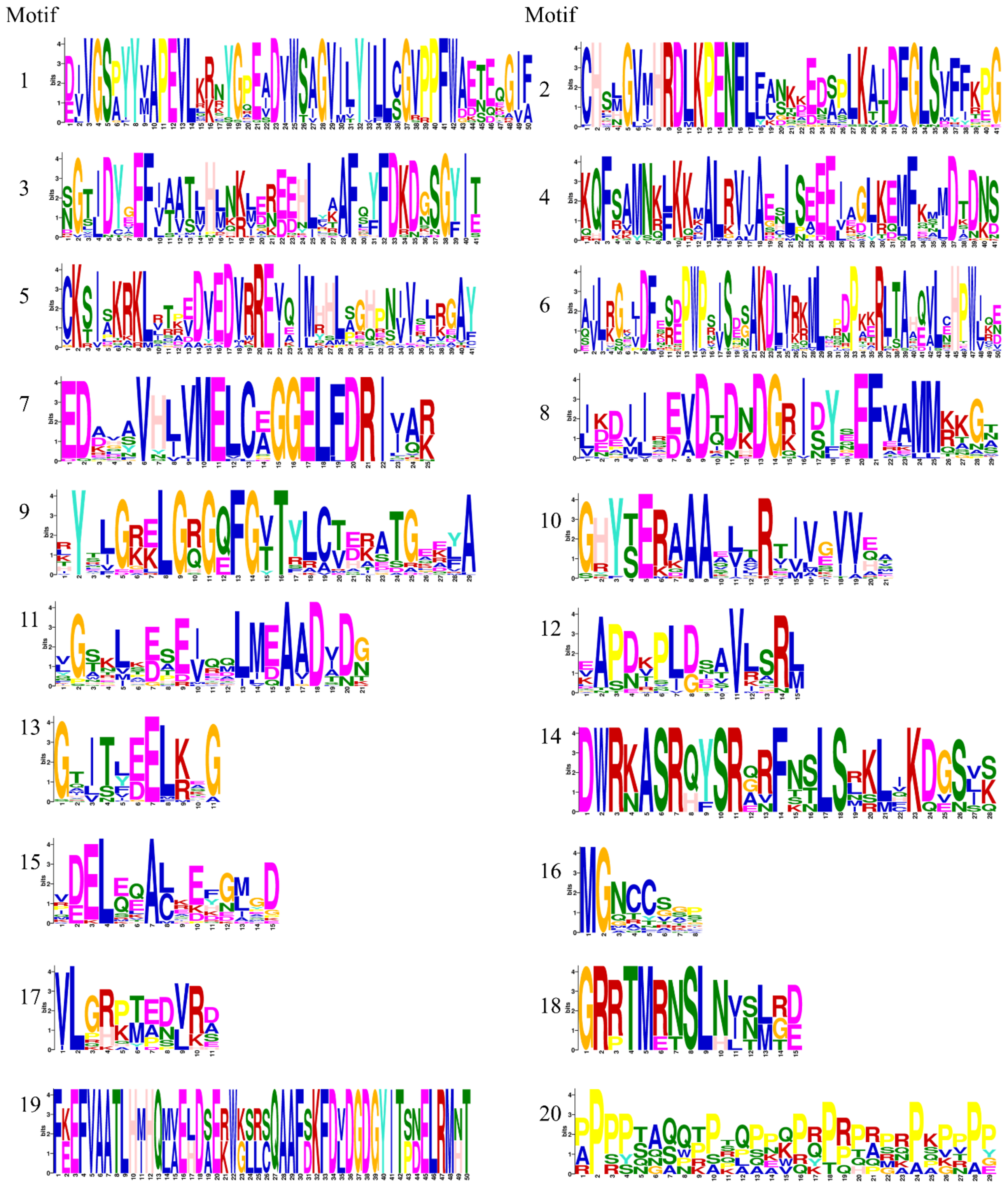

Fig. S4 Sequence logos for the 20 *CDPK*-associated motifs.

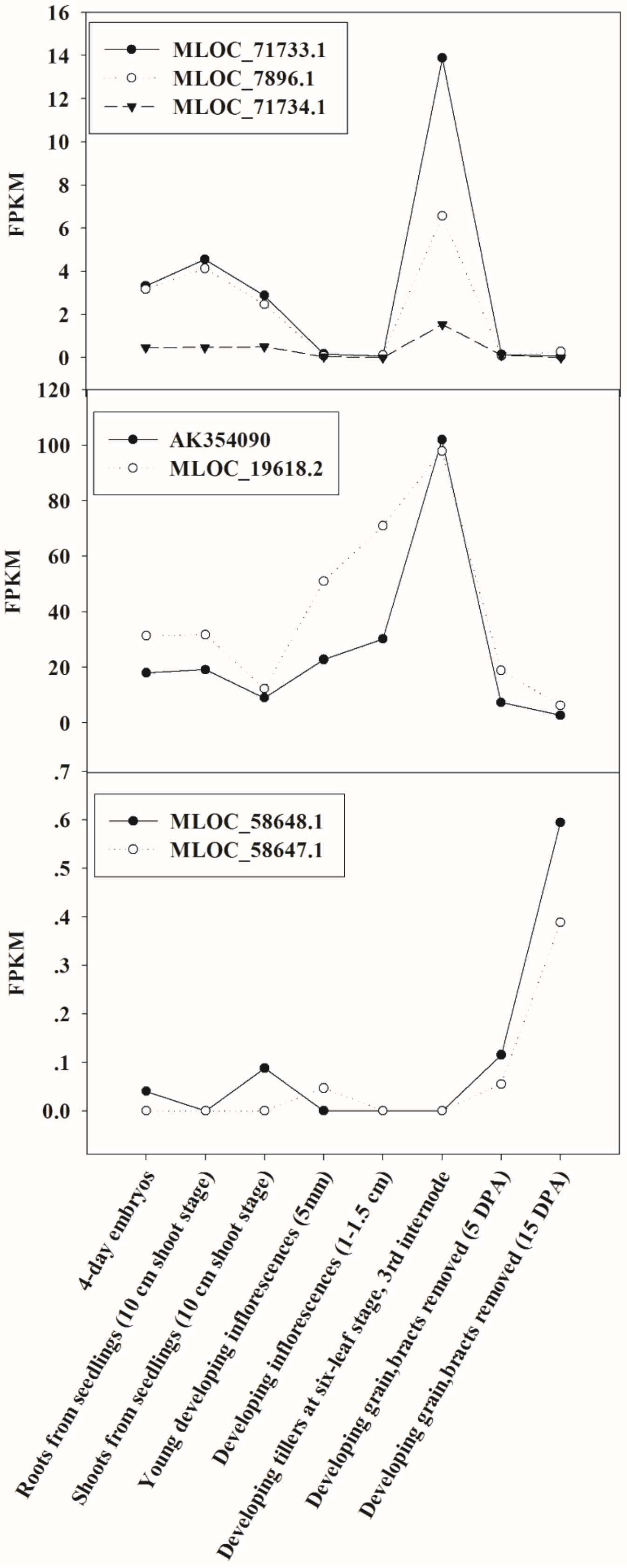

Fig. S5 Transcript levels of *MLOC_58648a*(a), *MLOC_19618a*(b), *MLOC_71733a*(c), *AK249361a* (d) and *MLOC_4965a*(e) with their EST sequences in different barley tissues and developmental stages.

Table S1. Primers used for full-length gene cloning.

Gene name	Locus ID	Forward primer for qRT-PCR (5'-3')	Reverse primer for qRT-PCR (5'-3')
HvCDPK16	MLOC_19618.2	ATGGACCGGGGCTCCAAGGAGCT	TCACTCGTTTCCCATCTTTACAG
HvCDPK9	MLOC_71733.1	ATGGGCAACGCGTGCTTCTCCTGCT	TCAGCGCGCCATGGAGATGGAG
HvCDPK7	AK249361.1	ATGGGTAATCAGAATGGGACC	TTAATGTACTTGAGCTGCATCTC
HvCDPK27	MLOC_58648.1	ATGGGCAACGTCTGCGTCGGC	TTAGCTATTTGACTTTATGTTCAAG
HvCDPK26	MLOC_4965.2	ATGGGCCAAAGGTGCTCCAAGG	CTATAGAACTAGATCTCTCCTCTTC

Table S2. Primers used for qPCR analysis.

Gene name	Locus ID	Forward primer for qRT-PCR (5'-3')	Reverse primer for qRT-PCR (5'-3')
HvCDPK1	MLOC_12765.1	GGCTGATGTTGATGGAAA	CTCCTGGTAGTTGATTCTTC
HvCDPK2	MLOC_54510.1	GGCTACATTTCCAAGGAG	GACTTCCGATACGATTTCC
HvCDPK3	AK371805	AGTTGACACCGATAAGGA	TGCTAAGGCTGTTGAATC
HvCDPK4	AK372866	CAACTCTCCACATACATCAG	GTATCCATCACCATCCAAAT
HvCDPK5	AK364859	TAGAGCGAGAGGAACATC	ATAATCAATGCGACCATCAT
HvCDPK6	MLOC_6934.1	GATCGACGACATCATCAG	TTCTTCATCATCGCAACAA
HvCDPK7	AK249361.1	AAGATGGCAGTGGCTATA	CTCTCAAGGTCACATTCAG
HvCDPK8	AK360154	TCTTCAGATGTTGATGGATG	TTATCTTCCCGTCCTTGT
HvCDPK9	MLOC_71733.1	TTCAAGAAGAAGGCGATG	CATGAAGGCGAACATCTC
HvCDPK10	MLOC_55774.3	GTACTTCGACAAGGACGGCA	TCGTTGTAGTCTATGCGCCC
HvCDPK11	MLOC_6391.1	CAGTGGTTACATTACTCAAGA	TTCCATCATTGTCCTTATCG
HvCDPK12	AK365481	ATTAGTGAGGCAGAGGTT	CATCATAGCAGTCAGGAATT
HvCDPK13	AK358395	CTATGGATGCCGATAACAG	CATAAGATCACGAATCTCAGTA
HvCDPK14	MLOC_76003.1	ACTACGACGAGTTCATCA	GTCCATCAACCCTTTCTC
HvCDPK15	AK363357	AGAGAAGGAAGACCACATAT	TGTATCCACTTCAGCAATG
HvCDPK16	MLOC_19618.2	GGTAGAGGAGCACTAGATTA	AATATGTCCTTCACCACTTC
HvCDPK17	AK373165	ATGGCAGTGGTTACATTAC	TCGGATAATATCGTCAAGATG
HvCDPK18	AK363630	TTGATGTTGACGGTGATG	AATATCAGCCTCCTCCAG
HvCDPK19	MLOC_79572.1	TGGCTTCATCACTAGAGAT	TCCTCGTAGTTAATCCTCC
HvCDPK20	AK365058	TTGTTACTCTGTCTGTTCAC	GCTTATCTTGCCATCCTTAT
HvCDPK21	AK374710	GAAGATGACCAACGACAA	TGAGGGTGTTGAAGTTTG
HvCDPK22	AK376018	AGTCAGAGATACAGATGCT	CAGGTAGGTACTCCTCATTA
HvCDPK24	AK373462	CAGCGGAACGATTGATTA	ATCCACTTCCATCCTTGT
HvCDPK25	MLOC_72770.1	GAGCAAGCCTTACAAGAG	GCCACGAACTCTGAATAG
HvCDPK26	MLOC_4965.2	GATTGATTGACTACGAGGAG	GAACTGGAATGCTGTGTAT
HvCDPK27	MLOC_58648.1	CAATGATGGACGCATAGAT	CCGACACTAAGGTTATACTG
HvCDPK28	MLOC_59921.1	CGGCTGATATTGACAACA	TCCCTCTCCAGTTTATTCA
HvCDPK29	MLOC_21560.3	AGTCGCAAGATGTTCAAG	CATCCCTTTGTCCTTTCC
Ubiquitin	GI:167075	TCTCGCCGACTACAACATCC	TTGTAGAACTGGAGGACGGC

Table S3. Primers used for *in situ* hybridization analysis.

Gene name	Locus ID	Forward primer for qRT-PCR (5'-3')	Reverse primer for qRT-PCR (5'-3')
HvCDPK1	MLOC_12765.1	ACACGGGGAGCAGTTTAAGG	TTTCCATCAACATCAGCCGC
HvCDPK16	MLOC_19618.2	CCTGGCCCCATGTTTCTGAT	CTCGAATAATGCCGCGAAGC
HvCDPK9	MLOC_71733.1	GAGCGGTTCACTGAGATCGT	CTCGGCATAGTCGAGGTAGC
HvCDPK10	NF	ATTCACCGACGTTGTCGGAA	TGTCGAAGTACTGGAACGCC
